# Selectins Mediate Small Cell Lung Cancer Systemic Metastasis

**DOI:** 10.1371/journal.pone.0092327

**Published:** 2014-04-03

**Authors:** Franziska Heidemann, Anna Schildt, Katharina Schmid, Oliver T. Bruns, Kristoffer Riecken, Caroline Jung, Harald Ittrich, Daniel Wicklein, Rudolph Reimer, Boris Fehse, Joerg Heeren, Georg Lüers, Udo Schumacher, Markus Heine

**Affiliations:** 1 Department of Anatomy and Experimental Morphology, University Medical Center Hamburg- Eppendorf, Hamburg, Germany; 2 Institut für Klinische Pathologie, Medizinische Universität Wien, Wien, Austria; 3 Heinrich-Pette-Institute for Experimental Virology and Immunology at the University of Hamburg, Hamburg, Germany; 4 Research Dept. Cell and Gene Therapy, Clinic for Stem Cell Transplantation, University Medical Center Hamburg- Eppendorf, Hamburg, Germany; 5 Department of Diagnostic and Interventional Radiology, University Hospital Hamburg-Eppendorf, Hamburg, Germany; 6 Department of Biochemistry and Molecular Cell Biology, University Medical Center Hamburg- Eppendorf, Hamburg, Germany; West German Cancer Center, Germany

## Abstract

Metastasis formation is the major reason for the extremely poor prognosis in small cell lung cancer (SCLC) patients. The molecular interaction partners regulating metastasis formation in SCLC are largely unidentified, however, from other tumor entities it is known that tumor cells use the adhesion molecules of the leukocyte adhesion cascade to attach to the endothelium at the site of the future metastasis. Using the human OH-1 SCLC line as a model, we found that these cells expressed E- and P-selectin binding sites, which could be in part attributed to the selectin binding carbohydrate motif sialyl Lewis A. In addition, protein backbones known to carry these glycotopes in other cell lines including PSGL-1, CD44 and CEA could be detected in *in vitro* and *in vivo* grown OH1 SCLC cells. By intravital microscopy of murine mesenterial vasculature we could capture SCLC cells while rolling along vessel walls demonstrating that SCLC cells mimic leukocyte rolling behavior in terms of selectin and selectin ligand interaction in vivo indicating that this mechanism might indeed be important for SCLC cells to seed distant metastases. Accordingly, formation of spontaneous distant metastases was reduced by 50% when OH-1 cells were xenografted into E-/P-selectin-deficient mice compared with wild type mice (p = 0.0181). However, as metastasis formation was not completely abrogated in selectin deficient mice, we concluded that this adhesion cascade is redundant and that other molecules of this cascade mediate metastasis formation as well. Using several of these adhesion molecules as interaction partners presumably make SCLC cells so highly metastatic.

## Introduction

Small cell lung cancer (SCLC) presently represents 13% of all lung cancer types and is the most aggressive of all lung tumor entities [1]. Due to the fast tumor doubling time and early haematogenous spread, the 5-year survival remains under 5% with a median survival rate of only a few months [2], [3]. SCLC typically metastasizes to brain, liver, bone marrow or adrenal glands. Because the formation of metastases is generally the leading cause for cancer death and based on the fact that therapeutic advances in SCLC did not strikingly increase the long-term survival of the patients, a more detailed insight in the metastatic cascade of SCLC is urgently required.

Metastasis - as a hallmark of cancer - is a multistep process starting with the uncontrolled growth of a primary tumor cell that overcomes the basement membrane and sends out angiogenic signals so that new blood vessels grow into the primary tumor cell mass [4], [5]. A subset of tumor cells detaches from the primary tumor and enters the circulation. The circulating tumor cells need to escape from the blood stream to invade the connective tissue of a distant organ. Therefore circulating tumor cells interact with the normal endothelium at the site of the target organ in a leukocyte-like manner. Once they have transmigrated the endothelium and have settled in the connective tissue stroma, tumor cells have to divide again in order to form a clinically detectable metastasis [6], [7].

Leukocytes use a cascade of cell adhesion molecules to attach and transmigrate endothelial cells in order to lodge into connective tissue stroma at the site of an inflammation. This adhesion cascade consists of a series of interrelated steps starting with tethering, followed by rolling, adhesion, intraluminal crawling and is finished by paracellular or transcellular migration of the endothelial cell [8]. The initial leukocyte rolling on the luminal surface of endothelial cells is mediated on the endothelial side by a class of carbohydrate binding proteins called E- and P- selectins. These two selectins bind to their carbohydrate ligands on the leukocytes in a Ca^2+^- dependent fashion. The carbohydrate determinant consists of sialyl Lewis^X^ or sialyl Lewis^A^ tetrasaccharides [9]. Known selectin ligand carrying protein backbones are PSGL-1, ESL-1 and CD44 [10]. In addition to leukocytes [11], circulating tumor cells have been shown to express the known selectin ligands [6], [7], [12]. For instance, the protein backbones PCLP-1 and CEA (CEACAM5) on colon and prostate cancer cells can be glycosylated with carbohydrate structures which bind to E-selectin [13], [14], [15].

The hypothesis that metastasis formation is mediated by selectins is supported by several spontaneous metastasis models of human tumor cells xenografted into immunodeficient mice. HT29 colon carcinoma cells [16] as well as DU4475 breast carcinoma cells [17] transplanted into E-/P- selectin deficient mice showed a significantly decreased number of spontaneous metastases in the lung compared with selectin-expressing wild type mice. It could also be demonstrated that peritoneal metastasis of pancreatic adenocarcinoma was reduced in E-/P- selectin deficient mice [18].

Recent investigations of the OH-1 cell line representing the classic SCLC phenotype [19] revealed a firm adhesion of OH-1 cells to an E-selectin fusion protein under physiological flow conditions. OH-1 cells displayed selectin binding sites as well as sialyl Lewis x and PSGL-1 [20]. Hence, our investigation focused on the influence of selectins in the development of metastases in SCLC and their potential selectin ligands on SCLC cells.

## Materials and Methods

### Cell line

The human small cell lung cancer cell line OH-1 was obtained from pleural effusion and kindly provided by Uwe Zangemeister-Wittke (University of Bern, Department of Pharmacology).

OH-1 cells were cultured *in vitro* using RPMI 1640 medium (Gibco/Life Technologies, Paisley, Scotland) complemented with 10% heat inactivated fetal bovine serum (FBS, Gibco), 2 mM L-glutamine (Gibco), 100 U/ml penicillin and 100 µg/ml streptomycin (Gibco) under standard cell culture condition (37°C, 100% relative humidity, 5% CO_2_).

### Lentiviral transduction

For bioluminescence imaging and intravital microscopy OH-1-LUC/mCherry cells expressing the luciferase from *Photinus pyralis* and the fluorescent protein mCherry were generated. For this purpose the Luc2 cDNA (Addgene Plasmid #24337) was cloned into the 3^rd^ generation HIV1 derived SIN vector LeGO-iC2-Puro^+^ [21]. Besides mCherry as marker gene, this lentiviral vector expresses Puromycin N-acetyl-transferase, conferring resistance to puromycin. Lentiviral particles were generated as described [22]. In brief, 293T cells were co-transfected with the vector plasmid LeGO-iC2-Puro^+^-Luc2 and the packaging plasmids phCMV-VSV-G, pMDLg/pRRE and pRSV-Rev by calcium phosphate precipitation. The supernatants containing lentiviral particles were collected 24 h after transfection. Functional titers were measured by FACS analysis 3 days after transduction of 293T cells. For lentiviral transduction of OH-1 cells 1×10^5^ cells/ml were plated in 24 well plates. The next day supernatants containing viral particles and 8 µg/ml Polybrene (Sigma) were added for 24 h. For the selection of transduced OH-1 cells, regular culture medium was supplemented with 2.5 µg/ml puromycin.

### Flow Cytometry

In order to evaluate binding sites and their protein backbones cells on the cell surface of OH-1 cells, cultured OH-1-LUC/mCherry cells were detached with Cell Dissociation Buffer (Gibco, Carlsbad, US) and stained for sialyl Lewis A (abcam, dilution 1∶1000), CEA (Cell Signalling, dilution 1∶200), CD44 (AbD Serotec, dilution 1∶1000), PSGL-1 (Santa Cruz, dilution 1∶200), E- and P-selectin binding sites themselves using E- and P-selectin fusion proteins (R&D Systems, dilution for E-selectin 1∶1000, for P-selectin 1∶100), EpCAM (Dako, dilution 1∶59), Muc18 (GeneTex, 1∶1800) and NCAM (R&D Systems, dilution 1∶1000). The corresponding isotype controls (IgG1 for PSGL-1, EpCAM, Muc18, CEA, E- and P-selectin; IgG2a for CD44 and NCAM; IgM for sialyl Lewis A) were incubated in parallel. The antibody expression was determined with FACS CALIBUR flow cytometer (Becton Dickinson, Heidelberg, Germany) and analyzed with Win MDI 2.9 software.

*In vivo* grown OH-1 cells from a primary OH-1-Luc/mCherry tumor were investigated by FACS analysis. Cells were double labelled for anti-CEA and a human E- or P-selectin fusion protein.

### Intravital confocal microscopy

For intravital microscopy, intestine was dissected in Ketamine/Xylazine (230 mg/kg and 20 mg/kg body weight) anaesthetized pfp/rag2 mice and mesenterial veins were visualized by a confocal microscope equipped with a resonant scanner (Nikon A1R). To induce the expression of E- and P-selectins on the apical surface of endothelial cells, mice received an i.p. injection of murine TNF-α three hours prior investigation. OH-1-LUC/mCherry cells were injected via a carotid vein catheter, and 30 confocal images per second were recorded. Simultaneously, mesenterial veins were observed and mCherry-labeled OH-1 cells visualized by fluorescence illumination and recorded for 15 min with plan fluo oil x40/1.3 NA objective lens and with the laser excitation wavelength of 561 nm and the emission filter of 595 nm for mCherry, and with the excitation wavelength of 638 nm and the emission filter of 640 nm for the reflection mode. Later, the acquired data was analyzed. Leukocytes were identified as non-fluorescent rolling objects, OH-1-LUC/mCherry cells as red fluorescent cells. Every rolling event of OH-1-LUC/mCherry cells during the recording was determined. In addition, the rolling velocity of leukocytes and tumor cells was calculated (NIS-Elements). Recordings are attached as supplementary video.

### Xenotransplantation of OH-1 and OH-1-LUC/mCherry cells in immunodeficient mice

All animals were maintained in a pathogen-free environment with filter top cages, fed with sterile water and food ad libitum and constantly observed and weighted. All manipulations were performed under aseptic conditions. For inoculation, OH-1 or OH-1-LUC/mCherry cells were trypsinated and viable cells (5×10^6^) were suspended in 1 ml of FCS-free cell culture medium. Each immunodeficient mouse (strains: pfp/rag2, E/P-selectin deficient pfp/rag2, SCID) received a 200 µl aliquot of this suspension injected subcutaneously between the scapulae while being sedated with CO_2_/O_2_- anesthesia. At the beginning of the experiment mice were aged between 15 and 27 weeks and weights ranged between 17.4 g and 32.4 g.

### Magnetic resonance imaging (MRI) and bioluminescence imaging (BLI) of primary tumors and spontaneous metastases

For detection of spontaneous distant metastases *in vivo*, OH-1-LUC/mCherry cells were subcutaneously injected into SCID mice (n = 3). White fur of SCID mice with a Balb/c background facilitated the detection of luminescence signal deriving from luciferase-positive OH-1-LUC/mCherry cells. MRI and BLI were performed 18 days after tumor cell implantation. For MR imaging a 7-Tesla Bruker Clinscan animal MRI scanner (Bruker BioSpin MRI GmbH, Ettlingen, Germany) as well as a two-dimensional turbo spin-echo (TSE) sequence (axial orientation of MR images) were used while regulating the murine body temperature with a thermocouple heating system. For bioluminescence imaging, luciferin (SIGMA-Aldrich) was injected intraperitoneally (150 mg Luciferin/kg body weight) and photon emission was measured with IVIS 200 system (Xenogen, CA, USA). On 54^th^ day after OH-1-LUC/mCherry cell injection, the primary tumor was surgically resected and subsequently processed for histology. On day 117 luminescence signals were measured *in vivo* a second time. Shortly afterwards the mice were finally anaesthetized with Ketamine/Xylazine (400 mg/kg and 35 mg/kg body weight) and primary tumor and organs (lung, heart, leg) were removed for *ex vivo* luminescence imaging and thereafter processed for histology. For all mentioned procedures except sacrificing, mice where anaesthetized with inhaled isoflurane (1.5–2%).

### Metastasizing of OH-1-LUC/mCherry and OH-1 cells in selectin-deficient and wild type mice

Immunodeficient pfp/rag2 mice and E/P-selectin deficient pfp/rag2 mice were used [17]. For the metastasizing of OH-1 cells 3 pfp/rag2 and 5 E/P-selectin deficient pfp/rag2 mice (27 to 32 weeks old) were used. The mice were sacrificed when they failed the UKCCCR health score system or tumors started to ulcerate. The primary tumors and lungs were excised and fixed in 4% formalin for further investigation.

For the metastasizing of OH-1-LUC/mCherry cells 20 pfp/rag2 and 20 E/P-selectin deficient pfp/rag2 mice (15 to 27 weeks old) were used. Between day 32 and 41 after OH-1-LUC/mCherry cell injection, the primary tumor was surgically resected and subsequently processed for histology. Animals lived until they failed the UKCCCR health score system and they were finally anaesthetized with Ketamine/Xylazine (400 mg/kg and 35 mg/kg body weight) and luciferin were i.p. applied. Lung, leg and heart were removed for *ex vivo* luminescence imaging and thereafter processed for immunohistochemistry.

### Histology

For hematoxylin/eosin (H&E) staining and immunohistochemistry, formalin-fixed tissues were embedded in paraffin wax according to standard laboratory procedures. Five µm thick sections were cut using a sledge microtome (Techno Med. GmbH, Bielefeld). Every 10th section of each lung was saved for H&E staining and additionally two series of 20 following sections out of the middle of each lung were retained for immunohistochemistry. H/E staining was performed according to standard laboratory protocol. Ten H/E stained sections out of the center of each lung were examined through light microscope at 400× magnification, whereas the slides were blinded for the examination. The quantitative assessment of lung metastases was performed as previously described [23].

### Immunohistochemistry

The paraffin sections were deparaffinized, rehydrated and treated for antigen retrieval. Antibodies used were as follows anti-EpCAM (clone MOC31, DakoCytomation, Carpinteria, USA), anti-CD44 (MCA2726T, AbD Serotec, Düsseldorf, Germany), anti-CEA (#2383, Cell Signalling, Beverly, MA, USA) and anti- PGP9.5 (Dako, Hamburg, Germany). For anti-EpCAM antibody staining, sections were incubated with Type XXIV Proteinase (Sigma, Aldrich, Steinheim, Germany). For anti-CD44 antibody and anti-CEA antibody staining were microwaved in 10 mM citrate buffer (pH 6.0). Heat-induced epitope retrieval for anti-PGP9.5 antibody staining was performed using Dako Target Retrieval Sol. S3307. After washing with TBS, non-specific binding was blocked by 30 minutes incubation with either 10% normal rabbit serum (Dako, X0902, Hamburg, Germany) for anti-CD44, anti-CEA and anti-EpCAM (Dako, Hamburg, Germany) or 10% swine serum for anti-PGP9.5 (Dako, Hamburg, Germany), respectively. The sections were incubated for 60 minutes at equal concentrations of the primary antibodies and corresponding isotype controls which served as negative controls: CD44 (dilution 1∶25) and its isotype control IgG_2_b, CEA (dilution 1∶100) and isotype control IgG_1_ mouse, EpCAM (1∶35) and isotype control IgG1 mouse as well as PGP9.5 (1∶200) and its isotype control rabbit IgG, respectively. All antibodies were diluted in Dako Antibody Diluent (S2022, S3022). After intensive washing in TBS, sections were treated with a biotinylated rabbit anti-mouse antibody for CD44, CEA and EpCAM or swine anti-rabbit antibody for PGP9.5 at a dilution of 1∶200 for 30 minutes, respectively, followed by incubation with avidin-alkaline phosphate complex (ABC Kit, Vectastain, Vector, Burlingame, CA) for 30 minutes. After further washings, the alkaline phosphatase activity was visualized using napthol-AS-bisphosphat as a substrate and New Fuchsin as chromogen. The sections were counterstained with Mayer's hemalum und mounted with Aquatex (Merck KGaA, Darmstadt, Germany). Sections were photographed using a photo microscope equipped with a digital camera (Axioplan 2 with MRC5 digital camera, Zeiss Göttingen, Germany).

### Tissue multi arrays of clincal samples

Tissue multi arrays (TMA) of SCLC primary tumors were provided by Katharina Schmid, Clinical Institute of Pathology, Medical University of Vienna, Austria. A total of 70 formalin-fixed, paraffin-embedded lung cancer tissues from patients who had undergone surgery were included in this study. Cases that had been selected between 1986 and 2005 included 43 male and 27 female patients. The average age of the patient cohort was 63±10.7 (range 35–92 years). Cases were classified according to the WHO Lung Cancer Classification 2004. The characteristics of the population under study are summarized in table 1.

**Table 1:** Characteristics of the patient cohort.

Number of cases	
	70
*Gender*	
Male/female	43/27
*Tumor stage (%)*	
pT1/pT2	35.7/52.9
pT3/pT4	1.4/10.0
*Nodal stage (%)*	
pN0/pN1–pN3	50.0/50.0
Tumor recurrence (%)	52,9
Census (%)	45,7
Disease-free survival (months)	29,3
Overall survival (months)	33,8

### Immunohistochemical evaluation of TMA

The CEA and CD44 expression levels were evaluated under a microscope (Zeiss, Axioplan 2, Göttingen, Germany) by two independent observers (MH and US) blinded to the clinical data. If no consensus was achieved in the first instance, cases were discussed until observers' agreement was reached. Staining levels were classified according to staining intensity (negative, moderate and strong staining). Specimens were considered positive if >50% of the tumor cells were moderately or intensely stained.

### Statistical analysis

Survival curves were constructed using the Kaplan–Meier method and compared by the log-rank test. A two-tailed P<0.05 was considered statistically significant. Statistical analyses were performed using the software GraphPad Prism (Graphpad Software, Inc., CA, USA.)

### Ethics Statement

The methodology for carrying out the animal experiments was consistent with the UKCCCR guidelines for the welfare of animals in cancer research. The experiment was supervised by the institutional animal welfare officer and approved by the local licensing authority (Behörde für Soziales, Familie, Gesundheit und Verbraucherschutz; Amt für Gesundheit und Verbraucherschutz, Hamburg, Germany, project no. 92/09).

## Results

### Adhesion molecule expression of OH-1 SCLC cells grown *in vitro*

**Figure 1:**
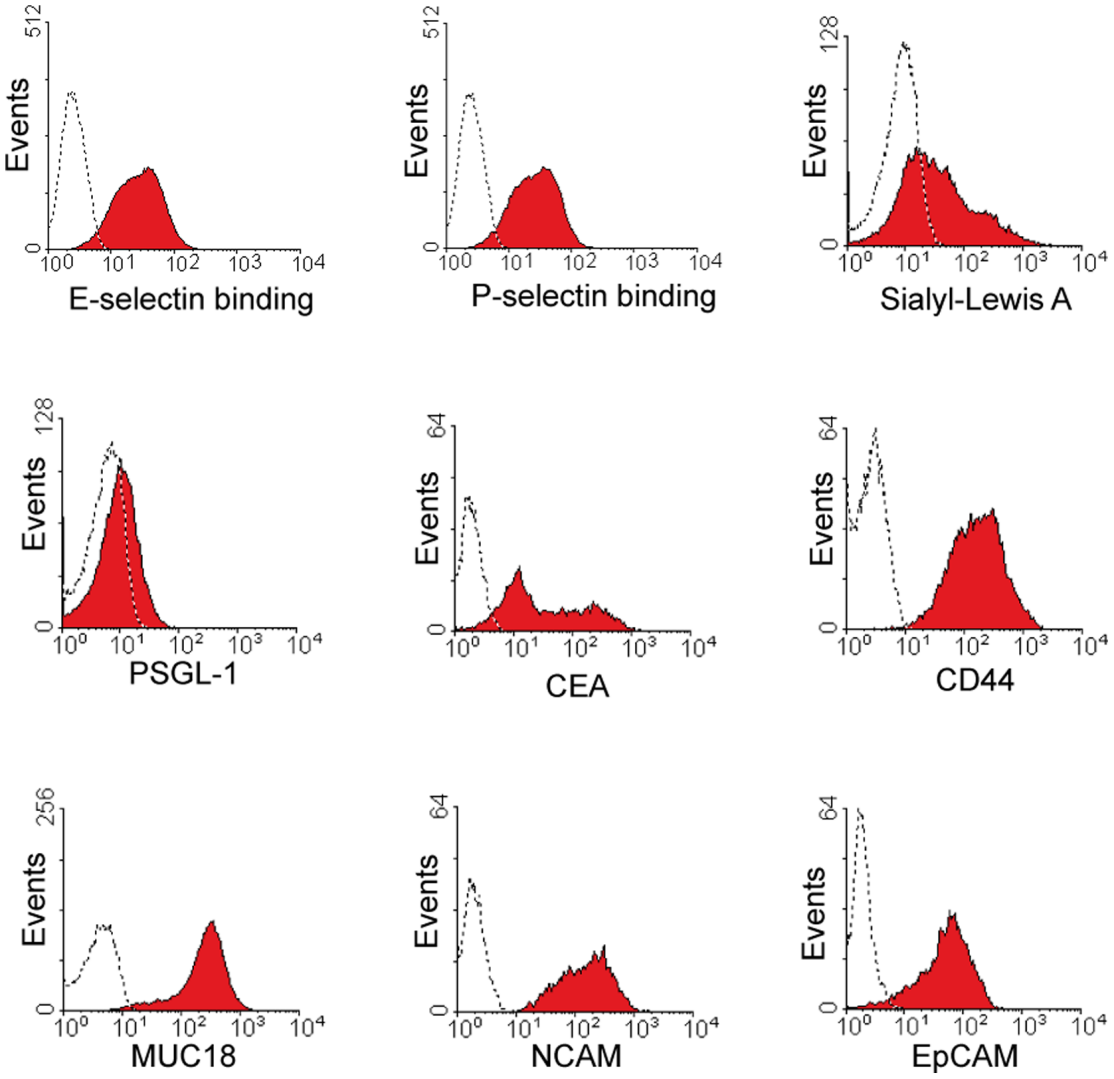
SCLC cell line OH-1 binds to selectins and expresses CEA. OH-1 cells, as a cell line of neuroectodermal origin, are positive for NCAM and EpCAM, which could be detected by flow cytometry analysis and can thus serve as a marker for these cells when grown in mice. Cells show binding to E- and P-selectin fusion protein. The glycosylation motif sialyl Lewis A, a binding partner recognized by selectins, could be detected with the CA19-9 antibody. The cell line OH-1 exhibits several known proteins which known to be protein backbones for selectin binding carbohydrate residues such as PSGL-1, MUC18, CD44 and CEA. Isotype controls are shown as dotted lines.

First we investigated the presence of E- and P-selectin binding sites, which were both present on OH-1 cells grown *in vitro* (Fig. 1). To further analyze the nature of these binding sites, we investigated for the presence of E- and P-selectin ligand sialyl Lewis A, which was also present. Next we analyzed for the protein backbone of known sialyl Lewis A and sialyl Lewis X carriers namely CEA, PSGL-1 and CD44, which were also present. Additionally, we examined OH-1 for further cell surface molecules known to be involved in metastasis in other cancer entities than SCLC. EpCAM, Muc18 as well as the neuronal adhesion molecule NCAM could also be detected at a high expression level.

### E- and P-selectin binding of SCLC cells grown *in vivo*

**Figure 2:**
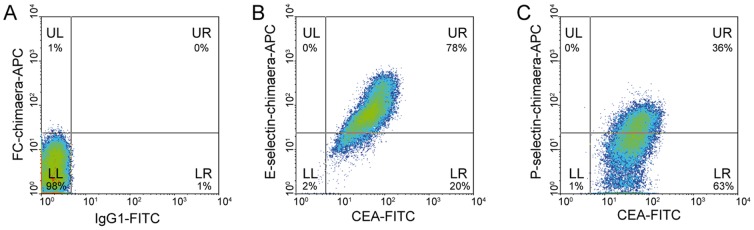
OH-1-LUC/mCherry cells grown in vivo are CEA and E-/P- selectin binding positive. Isolated OH-1-LUC/mCherry cells of primary tumors were stained in parallel against CEA and E- or P-selectin binding and FACS analyzed. (A) OH-1-LUC/mCherry cells showed no binding to isotype and FC-chimaera controls. (B) Almost all cells were CEA and E-selectin binding positive, by contrast (C) two thirds of the cells were P-selectin binding negative. Isotype controls are shown as dotted lines.

By use of FACS analysis (Fig. 2) we investigated whether *in vivo* grown OH-1 cells of primary xenografted tumors have maintained the ability to bind to selectins fusion proteins. Cells harvested from primary OH-1-LUC/mCherry tumors were double labelled with anti-CEA and a human E- or P-selectin chimaera. Almost all cells (78%) were both positive for anti-CEA staining and E-selectin binding (Fig. 2). Double staining of anti-CEA and human P-selectin chimaera revealed that 36% of the cells were both positive for P-selectin binding and CEA (Fig. 2C).

### *In vivo* behavior of circulating SCLC cells

**Figure 3:**
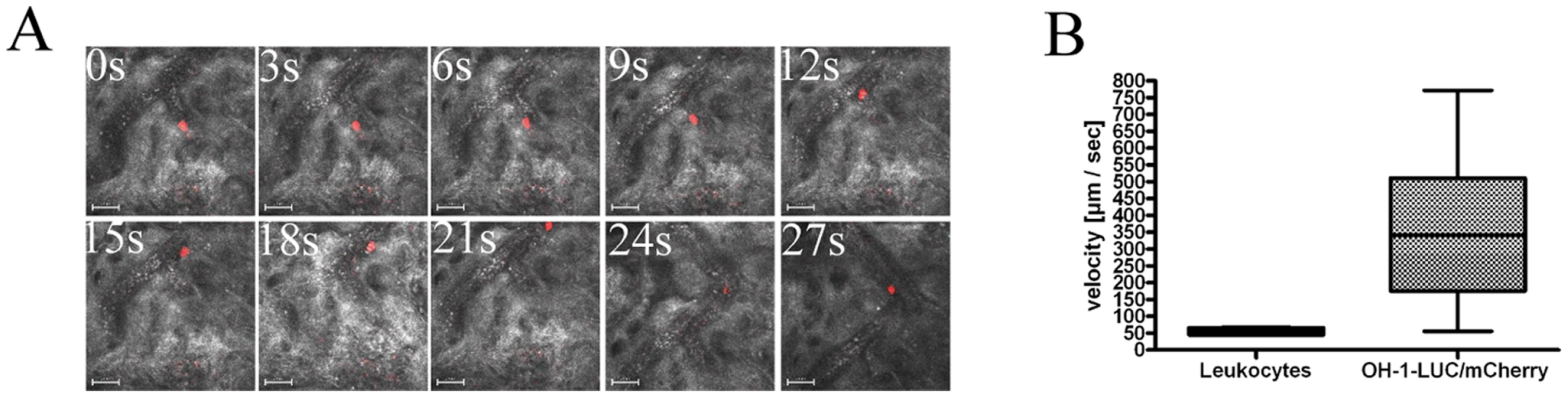
SCLC cells behave similar to leukocytes in murine mesenteric veins. Rolling behavior of injected fluorescent OH-1-LUC/mCherry cells (mCherry, red) in mesenteric veins (reflection mode, grey) were visualized in real time with high speed confocal intravital imaging. (A) The rolling behavior of one OH-1-LUC/mCherry cell were observed over 27 seconds. (B) Mean rolling velocity of injected OH-1-LUC/mCherry cells and endogenous leukocytes in mesenteric veins were determined with high speed intravital imaging (Nikon A1R confocal microscope). Scale bar: 500 µm for (F) and (H), 50 µm for (G).

OH-1-LUC/mCherry cells rolling on TNF-α treated vessel walls like leukocytes were identified using fast intravital confocal microscopy (figure 3A and Movie S1). As we also found rolling leukocytes, the rolling velocity of both cell types were measured. Leukocytes showed a mean rolling velocity of approximately 50 µm/sec and OH-1-LUC/mCherry cells of 350 µm/sec (Fig. 3B). Thus, the mean rolling velocity of OH-1-LUC/mCherry cells was approximately seven times faster than that of leukocytes, indicating that SCLC cells mimic leukocytes rolling behavior, however, to a lesser adhering efficiency.

### SCLC metastatic sites in immunodeficient mice

**Figure 4:**
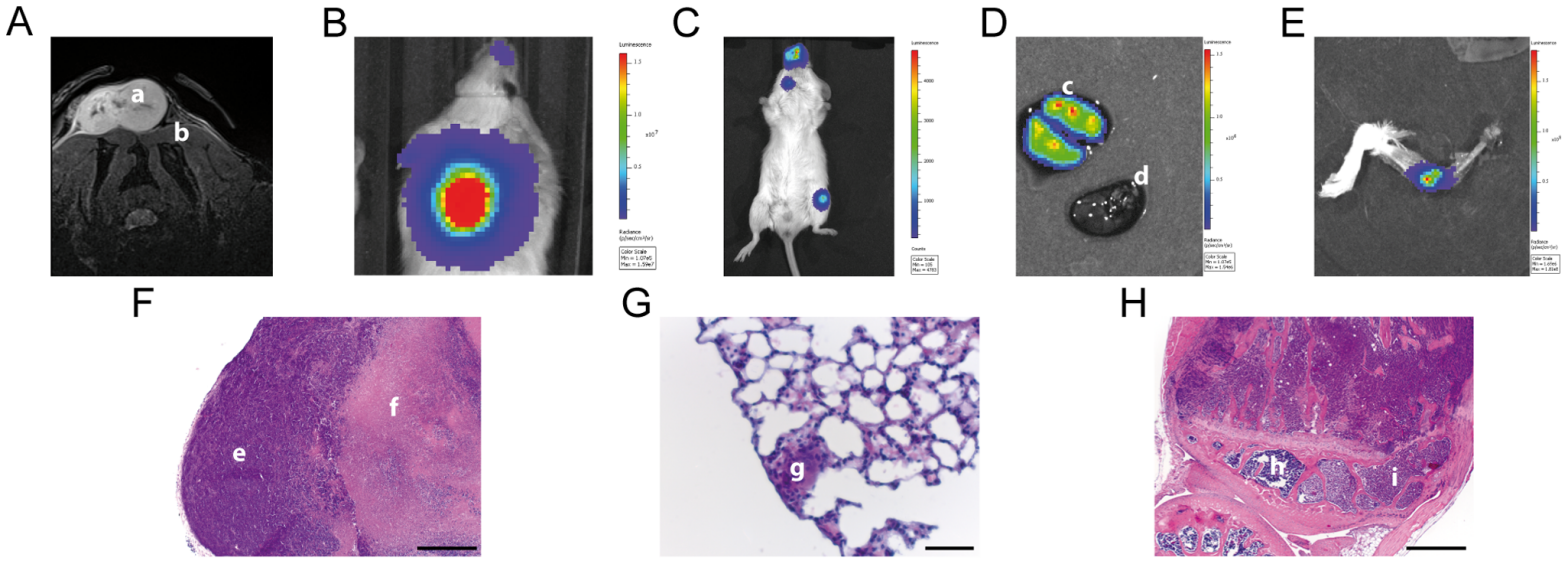
SCLC cell line OH-1 spontaneously metastasizes to lung and bone in immunodeficient mice. OH-1-LUC/mCherry cells were subcutaneously implanted (A) and grown as a primary tumor (a) for 18 days at the site between the scapulae (b) with a hyper-intensive appearance in a two-dimensional turbo spin-echo (TSE) sequence (MR images in axial orientation) and a positive bioluminescence signal (B) of the corresponding OH-1-LUC/mCherry tumor as in panel A. The primary tumor was surgically removed 51 days after OH-1-LUC/mCherry cell injection. (F) Corresponding paraffin sections of the resected tumor revealed living tumor cells (e) around a central necrosis (f). (C) Luminescence signals of metastasizing OH-1-LUC/mCherry cells in vivo were detectable in the areas of snout, distal femur and chest 117 days after injection. Organs were removed and bioluminescent signals were confirmed ex vivo. (D) The lungs showed several luminescence spots (c), while the heart (d) did not show any sign of luminescence. (G) H&E staining of corresponding lung sections showed a a small metastatic deposit surrounded by normal lung tissue (g). (E) Luminescence from the tibia at the knee joint could be detected and (H) H&E staining of a corresponding bone paraffin section showed metastasized OH-1-LUC/mCherry cells (i) next to healthy bone marrow (h). Scale bars: 500 µm for (F) and (H), 50 µm for (G).

To investigate the spontaneous metastasis site of SCLC cells OH-1-LUC/mCherry cells were subcutaneously injected in SCID mice as a xenograft. In order to non-invasively verify OH-1-LUC/mCherry primary *in vivo* tumor growth, MR and bioluminescence imaging were carried out. On day 18 after OH-1-LUC/mCherry inoculation the existence of the OH-1-LUC/mCherry primary tumor, which appeared as a hyper intensive lesion in T2-weighted axial MR images, was demonstrated at the inoculation site located between the scapulae (Fig. 4A). The corresponding bioluminescence images displayed luminescence signals derived from the OH-1-LUC/mCherry tumors (Fig. 4B). As the primary tumor growth was too fast to locate the small metastases in a non-invasive way, the primary tumor was resected on day 54 after OH-1 inoculation and 63 days later the mice were reinvestigated for *in vivo* luminescence. Luminescence signals could be detected in the area of the snout, upper chest and left hind leg (Fig. 4C). To validate the presence of metastases the tissues at these sites were excised after sacrificing of the animals and the bioluminescence was reinvestigated *ex vivo*. Intensive signals could be verified in the lungs (Fig. 4D) and knee joint (Fig. 4E) whereas the heart did not show any signal (Fig. 4D). To verify that bioluminescent areas were indeed metastases, histological examination was carried out. Vital tumor cells in the OH-1-LUC/mCherry tumor were located around a central necrosis (Fig. 4F). Lung and bone metastases could be confirmed adjacent to healthy tissues.

### Role of E- and P-selectin for metastasis *in vivo*

Since cells of the CEA positive SCLC cell line OH-1-LUC/mCherry cells successfully metastasized in immunodeficient mice we investigated the role of E-/P-selectins for this process *in vivo*.

**Figure 5:**
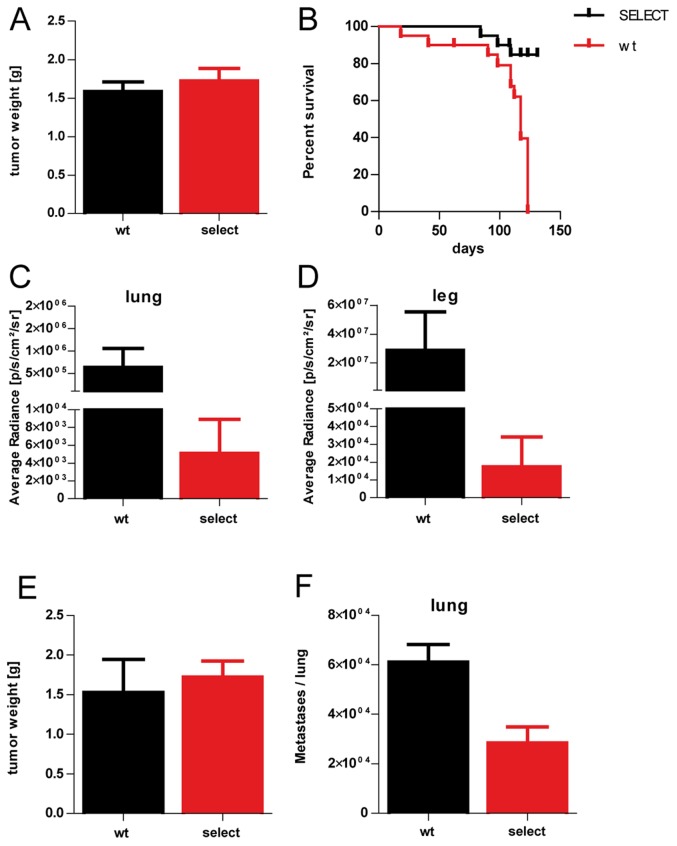
Metastasis rate of SCLC cells is reduced in E-/P- selectin knockout mice. OH-1-LUC/mCherry cells were subcutaneously injected in E-/P-selectin double knockout mice (select) or wild type litter mice (wt) and resulting tumors grown for 31–41 days. Tumors were resected and weighted. (A) No statistical difference concerning tumor weight could be determined (Students t test, p = 0.5025). Tumor resected mice lived until they reached end point criteria. (B) Kaplan Meier survival curve shows significantly decreased median survival of wt (n = 20) mice compared with select (n = 20) mice (log-rank test, p<0.0001). The organs were removed and a significant difference comparing wt (n = 12) and select mice (n = 18) were determined in terms of bioluminescence of the removed lungs (C) and legs (D) (Mann-Whitney test, p = 0.0003). In a parallel approach OH-1 cells were subcutaneously injected in select (n = 5) or wt (n = 3) mice and tumors grown for 36–65 days. Organs were removed and the tumor weight was determined. There was no statistically significant difference in the weight of the primary OH-1 tumors between mouse strains (E) (students t test, p = 0.6489). The numbers of spontaneous lung metastases in select and wt mice were determined. Note that the number of metastases was statistically significantly reduced by 50% in select mice in comparison with wild type mice (F) (students t test, p = 0.0181).

OH-1-LUC/mCherry cells were subcutaneously injected into wild type (wt) and E-/P-selectin-deficient knockout (select) mice. Resulting tumors were resected after 31–41 days and their weight was determined (Fig. 5A). We could not detect any statistically significant difference between tumors grown in wt and select mice, indicating that the selectin status of the immunodeficient mice did not directly influence tumor cell proliferation. Tumor resected mice lived until they reached the termination criteria. Kaplan Meier survival curve revealed a significantly decreased median survival of wt (n = 20) mice compared with select (n = 20) mice (log-rank test, p<0.0001) (Fig. 5B and table 2). Lungs and legs were removed and analyzed separately for bioluminescence intensity. We could show a significant difference between organs from wt (n = 12) and select mice (n = 18) with reduced bioluminescence signal within the lungs (Fig. 5C) and legs (Fig. 5D) from the selectin-deficient mice compared to the wt mice indicating a considerably reduced metastatic load in the selectin deficient mice (Mann-Whitney test, p = 0.0003).

**Table 2:** Summary of all cases included in the Kaplan-Meier analysis of wild type (wt) against E-/P-selectin knockout (select) mice.

Mouse Strain	No. Deaths/No. Patients	Censored Subjects	Median Survival [months]	Compared with	Hazard Ratio (95% CI)	p
wt	18	2	117			
select	3	17	Undefined	wt	0.1174 (0.04590 to 0.3002)	<0.0001

To exclude the influence of lentiviral transduction on the metastasis rate we analyzed the parental OH-1 cell line in a parallel approach. OH-1 cells were subcutaneously injected in select (n = 5) or wt (n = 3) mice and tumors grown for 36–65 days. Lungs and primary tumors were removed and the tumor weight was determined. The average tumor weight (Fig. 5E) was 1,53 g (n = 3) in wild-type and 1,72 g (n = 5) in selectin- double-deficient mice. There was no statistically significant difference in the weight of the primary OH-1 tumors between mouse strains (students t test, p = 0.6489). Moreover, all mice developed spontaneous micrometastases to the lung. However, the number of metastases to the lungs significantly differed between wild type and the E/P-selectin-deficient mouse strain. The number of metastases ranged in wild type mice ranged between 47560 and 72165 (mean: 61076) and in E/P-selectin double-deficient mice metastases ranged between 16790 and 53317 (mean: 28446) (Fig. 5F). Note that the absence of E- and P- selectins decreased the number of spontaneously developed lung metastases by 50% (students t test, p = 0.0181).

### Immunohistochemical detection of selectin ligand protein backbones of SCLC cells *in vivo*

**Figure 6:**
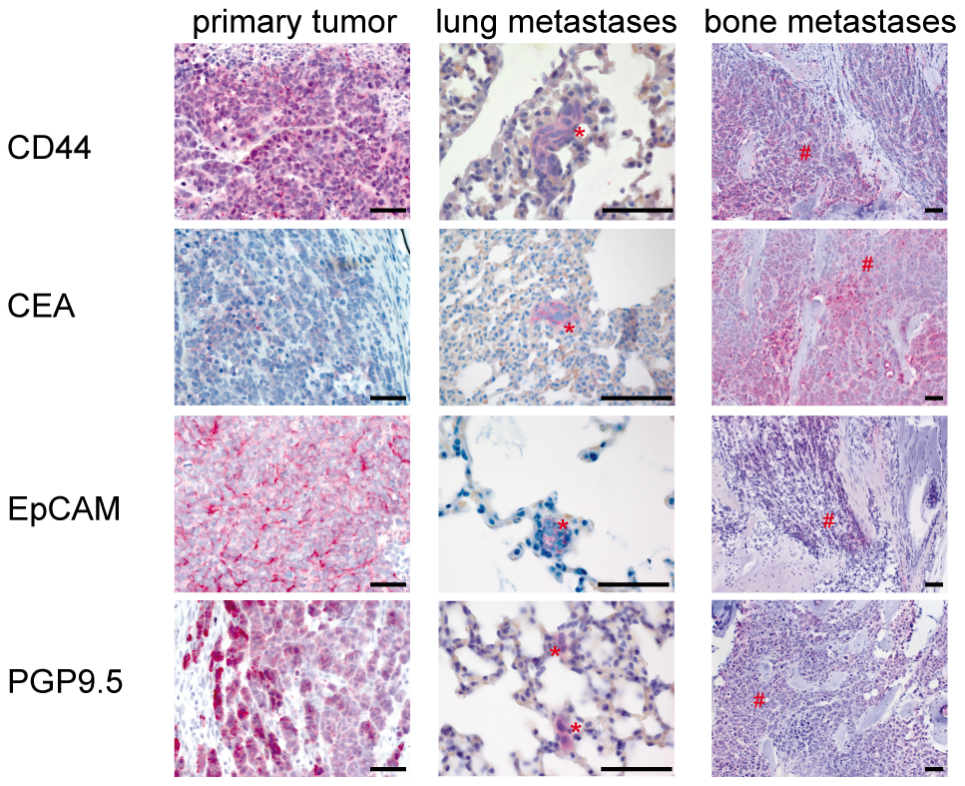
Spontaneous OH-1 metastases express CEA. Primary OH-1 tumor as well as spontaneous OH-1 lung and bone metastases were positively stained for CD44, CEA, EpCAM and PGP9.5 (red = positive cells). Metastases are indicated by red asterisks. Scale bar: 50 µm for all panels.

To investigate that selectin ligands and further cell surface molecules were expressed *in vivo* in primary tumors and metastases we have analyzed their expression by immunohistochemistry (Fig. 6). The established selectin ligand CEA could be detected in primary tumors and even observed with an increased expression in lung and bone metastases. CD44 and EpCAM, known to be present in a variety of tumor entities, were also expressed in tumor and lung metastases to a high degree. In addition, the SCLC marker protein PGP 9.5, also known as UCH-L1, was abundantly expressed in cells of the primary tumor, lung and bone metastases.

### Clinical relevance of tumor CEA and CD44 expression for the overall survival of SCLC patients

**Figure 7:**
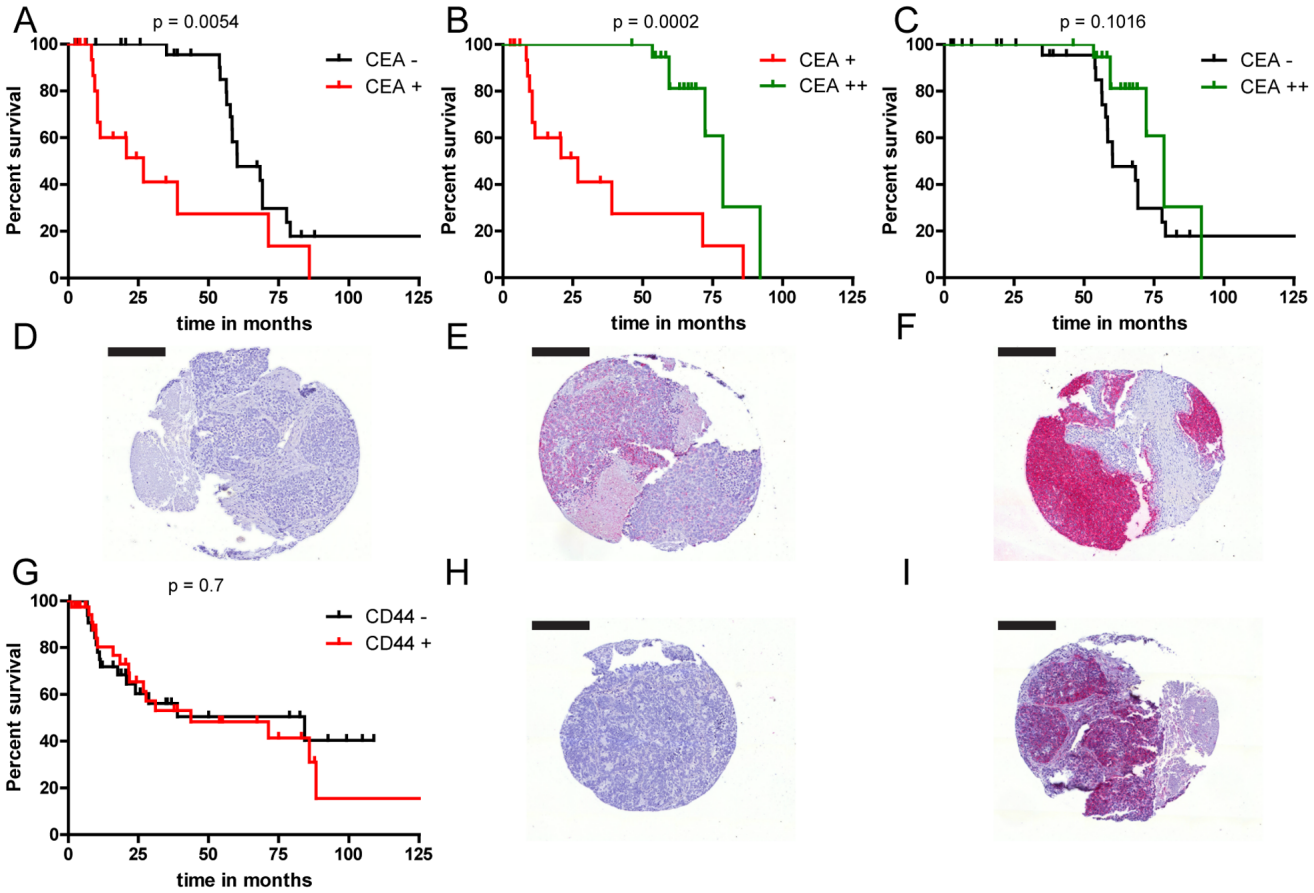
Decreased survival of SCLC patients with moderate CEA expression levels. TMA of SCLC patients was immunohistochemically stained against CEA. Three patient groups were discriminated: (D) no CEA expression (CEA−), (E) moderate CEA expression (CEA+) and (F) strong CEA expression (CEA++). Kaplan Meier survival curves show significantly decreased median survival of patients with moderate CEA levels compared to patients with (A) negative (log-rank test, p = 0.0054) or (B) strong CEA levels (log-rank test, p = 0.0002), but no significant difference between median survival of patients (C) with negative compared to strong CEA levels (log-rank test, p = 0.1016). In parallel a TMA of SCLC was immunohistochemically stained against CD44. Two patient groups were discriminated: (H) CD44 negative (CD44−) and (I) CD44 positive (CD44+). Kaplan Meier survival curve shows no statistical different median survival of patients (G) with negative CD44 levels compared with patients with positive CD44 levels (log-rank test, p = 0.7). Scale bar = 200 µm.

In order to estimate whether our data on expression of CEA are relevant for interpretation of CEA levels as a prognostic marker, we have analyzed a tissue multi array (TMA) of lung cancer biopsies representing 70 patients. Expression levels CEA and CD44 were analyzed and correlated with survival rates of the patients. Positive CEA expression was detected in 56% (39/70) and CD44 expression in 54% (38/70) of patient samples analyzed (table 1). CEA staining was further classified into no (n = 31), moderate (n = 19) and strong CEA (n = 20) expression subgroups (Fig. 7D, E, F and table 3). Patients with moderate CEA expression had a significantly shorter overall survival than patients with no CEA expression (hazard ratio for death, 0.2525; 95% confidence interval, 0.09580–0.6657; P = 0.0054) (Fig.7A). Surprisingly, the overall survival of patients with strong CEA expression was also significantly different from patients with moderate CEA expression (hazard ratio for death, 8.176; 95% confidence interval, 2.658–25.15; P = 0.0002) (Fig. 7B). No significant difference was determined concerning overall survival of patients with no and strong CEA expression (hazard ratio for death, 2.073; 95% confidence interval, 0.4352–1.748; P = 0.1016) (Fig. 7C).

**Table 3:** Summary of all cases included in the Kaplan-Meier analysis of CEA and CD44.

Biomarker	No. Deaths/No. Patients	Censored Subjects	Median Survival [months]	Compared with	Hazard Ratio (95% CI)	p
CEA staining						
Negative (−)	15	16	60			
Moderate (+)	11	8	27	Negative	0.2525 (0.09580 to 0.6657)	0.0054
				Strong	8.176 (2.658 to 25.15)	0,0002
Strong (++)	6	14	79	Negative	2.073 (0.8661 to 4.964)	0,1016
CD44 staining						
Negative (−)	15	18	84			
Strong (+)	17	21	44	Negative	0.8722 (0.4352 to 1.748)	0.6997

In contrast, no significant association between CD44 expression and overall survival (Fig. 7H, I and table 3) was detected.

## Discussion

The bleak outlook for patients with SCLC is due to the fact that this malignancy has the propensity to metastasize early and once widespread formation of metastases has occurred no cure is possible. We hence focused our efforts on elucidating the molecular mechanism of SCLC metastasis formation. As metastasis formation is a complex process, it can only be analyzed *in vivo*. We therefore used the established human SCLC cell subcutaneous implantation into pfp/rag2 mouse model for analyzing the metastatic behavior of SCLC cells [24]. From a functional point of view, we focused on the role of E- and P-selectins as mediators of metastases for several reasons. First of all, E- and P-selectin have been shown to be of importance in breast and colon cancer metastasis formation *in vivo* [16], [17]. Using a *in vitro* flow assay, Richter et al. could show that E-selectin binding in this assay correlated well with the metastatic behavior of the SCLC cells in pfp/rag2 mice, i.e. cell lines with a higher number of adhesive events also showed a higher number of spontaneous lung metastases than those with lower number of adhesive events [20]. We therefore investigated the metastatic pattern of human SCLC cell lines in E- and P-selectin deficient pfp/rag2 mice in comparison to wild type pfp/rag2 mice in order to elucidate the role of these two selectins in the metastatic progression of SCLC.

Initially, to investigate *in vivo* tumor growth and metastatic pattern of SCLC cells, OH-1-LUC/mCherry cells were implanted subcutaneously into immunodeficient mice. OH-1-LUC/mCherry cells spontaneously metastasized to the lungs and the bone marrow of the tibia and femur, typical sites of metastatic deposits of SCLC patients [25]. It is known that E- and P-selectins mediate leukocyte adhesion and diapedesis and tumor cells are suspected to use a similar mechanism to metastasize to distant sites as proven in several xenograft models of metastasis formation [8], [13], [14], [15], [26]. Accordingly OH-1-LUC/mCherry cells were xenografted into E-/P-deficient mice. The E-/P-selectin deficiency led to a reduction of spontaneous SCLC cells metastases by 50% as compared to wildtyp pfp/rag2 mice thereby corroborating finding of Köhler et al. for colon carcinoma and Stübke et al. for breast carcinoma [16] [17]. In addition to the higher number of spontaneous lung metastases the median survival time of wild type mice in our xenograft model was significantly reduced in comparison to E- and P-selectin deficient animals further stressing the roles of E- and P-selectin interactions with tumor cells for the clinical outcome of this disease as well.

As we have now shown that E-and P-selectin play a functional role in metastasis formation in SCLC, we therefore wanted to analyze the *in vivo* behavior of SCLC cells in order to observe if they behave in terms of rolling and tethering also similar to leukocytes. By observing single circulating OH-1 cells in murine veins using fast intravital confocal microscopy, we analyzed the flow characteristics of tumor cells within the capillary system. OH-1 cells expressing the red fluorescent protein mCherry and the bioluminescence enzyme luciferase (OH-1-LUC/mCherry) were injected via a carotid catheter into the blood stream and their interactions with the endothelium of mesenterial veins were recorded. The tumor cells interacted with endothelial cells in a way similar to leukocytes, but to a lesser extent. The higher rolling velocity could be partly explained by findings of McEver et al. [11] on cell tethering and rolling under flow conditions. At a given shear stress, larger cells such as OH-1 cells possess higher rolling velocities because they are located more centrally in the vessels compared with leukocytes. Also, a higher velocity produces more collisions with the vessel walls but reduces the contact time between receptor and ligands. Furthermore, Sundd et al. [27] and Wan et al. [28] state that leukocyte rolling in postcapillary colonic venules is predominantly mediated by interaction of P-selectin and PSGL-1. As PSGL-1 is the dominant ligand on leukocytes for P-selectin [11] and our FACS analysis showed only few PSGL-1 on OH-1, this might be another reason for lower binding capacity of OH-1 cells. Thus, higher rolling velocity of OH-1 cells could also be explained because tumor cells did not have the same composition of adhesion molecules required equipment compared with leukocytes [7], [29].

Our next aim was to determine potential selectin ligands under controlled conditions. We analyzed the human SCLC cell line OH-1 by flow cytometry for the expression of CEA and other adhesion molecules known to play a role in haematogenous metastasis. OH-1 cells expressed the known SCLC marker proteins EpCAM, PGP9.5, MUC18 and NCAM and were furthermore labeled with antibodies directed against CEA, PSGL-1 and CD44 which can (properly glycosylated) function as selectin ligands [12], [13]. Accordingly OH-1 cells expressed the selectin ligand sialyl Lewis A and additionally displayed E- and P-selectin binding sites providing the possibility for a similar behavior *in vivo*. Correspondingly, *in vivo* grown cells retained the feature of E- and P-selectin binding and were positive for CEA expression. Note that *in vitro* grown cells showed different CEA populations in FACS analysis compared to *in vivo* grown ones. *In vivo* grown cells with low CEA expression showed also lesser E-selectin binding compared to cells with high CEA expression indicating a direct link between CEA expression level and selectin binding.

The expression of CD44 and CEA as markers of prognosis in several types of cancer is well known [30]. However, their direct role in the metastasis cascade of tumor cells remained unclear so far. Much research has been done on CD44 expression in lung cancer. However, staining for CD44 or splice variants such as CD44v6 were often found to be negative in small cell lung cancer [31], [32], [33], [34], [35]. Other investigations showed CD44 positivity in 16%, 36% or 57% of SCLC samples [36], [37], [38]. In our SCLC patient cohort 54% of all samples were CD44 positive. However, no correlation between CD44 expression in SCLC and prognosis has been reported so far. The rare numbers of CD44 positive SCLC tumors might be one reason. Although CD44 is believed to be associated with increased metastatic potential in several other cancer entities, we also could not find evidence for a prognostic value of CD44 tissue expression in SCLC.

To determine whether CD44 or CEA expression of tumor cells is an important factor for the overall survival of SCLC patients, we used tumor micro arrays (TMA) of clinical biopsies. Each TMA consisted of 70 SCLC patients and was immunohistochemically stained against either CEA or CD44. The patients were grouped according to their expression levels and subsequent Kaplan-Maier analyses were done. We could show that patients with moderate CEA expression had a significantly shorter overall survival compared with patients with negative or strong CEA expression. One reason for short lifetime of SCLC patients after diagnosis is the high metastasis rate of the tumor. We conclude that SCLC cells expressing moderate levels of CEA have an unknown advantage in steps of metastasis. It is known that CEA molecules can interact in a homophilic manner [39], [40]. Probably high CEA expression on tumor cells leads to more homophilic interaction so that a saturation of CEA binding capacity inhibits the possibility to extravasate via selectin interaction. CEA-expressing tumor cells are more likely to resist immunosurveillance by interacting with NK cell receptor CEACAM-1 [41]. This might on one hand be an advantage for survival within the blood stream but could also be a disadvantage because of the lower binding capacity of CEA to selectins. Sialyl Lewis x and sialyl Lewis A are the carbohydrate determinants needed for selectin binding. These carbohydrates are linked to glycoconjugates in Golgi compartment through activity of N-acetylglucosaminyl-, galactosyl-, sialyl- and fucosyltransferases [42]. Thomas et al. provided evidence that CEA of the colon cancer cell line LS147T is a functional E-selectin ligand and knock down of CEA leads to loss of E-selectin binding [12]. McDermott et al. could show that overexpression of another E-selectin ligand MUC1 leads to loss of E-selectin binding which could partly be explained by oversaturated Golgi glycosylation machinery [43]. This could be an explanation of the phenomenon that patients with high levels of CEA expression have an equal long survival time as patients with no expression of CEA. We therefore hypothesize that only moderate levels of CEA are glycosylated in the way that they could serve as binding partners for selectins.

In summary, we could show that SCLC metastasizes in a selectin-dependent manner and SCLC cells possess various ligands for selectin interaction, including CEA. Moreover, selectins are important players in metastasis, but as metastasis is only reduced and not abrogated in E- and P- selectin deficient mice, other members of the leukocyte adhesion cascade like CD24, N-cadherin or Lamp1/2 must play a role as well [7]. Hence different tumor cells use different adhesion mechanisms to metastasize and therefore the molecules of the leukocyte adhesion cascade highjacked by SCLC cells are redundant. Presumably this redundancy leads to the highly efficient metastasis formation in SCLC.

## Supporting Information

Movie S1 **OH-1-LUC/mCherry cells rolling on TNF-α treated mesenteric vessel walls recorded by intravital microscopy.**
(MP4)
